# Social ties, employment and mental health in people with disabilities in Korea: evidence from a 2016–2018 panel survey

**DOI:** 10.1093/inthealth/ihaf078

**Published:** 2025-10-17

**Authors:** Jae-Hyun Kim, Hyun Jun Lee, Gyeong Min Lee, Youngsoo Kim

**Affiliations:** Department of Health Administration, College of Health Science, Dankook University 119, Dandae-ro, Dongnam-gu, Cheonan-si, Chungcheongnam-do, 31116, Republic of Korea; Dankook Institute for Health and Medical Policy, Dankook University, Cheonan, Republic of Korea; Department of Health Administration, College of Health Science, Dankook University 119, Dandae-ro, Dongnam-gu, Cheonan-si, Chungcheongnam-do, 31116, Republic of Korea; Dankook Institute for Health and Medical Policy, Dankook University, Cheonan, Republic of Korea; Department of Preventive Medicine, Gyeongsang National University, Jinju, Republic of Korea; Department of Public Health, Gyeongsang National University Changwon Hospital, Changwon, Republic of Korea

**Keywords:** disabilities, interpersonal relationships, mental health, social connectedness, subjective health

## Abstract

**Background:**

Social connectedness is a critical factor influencing health outcomes, particularly among people with disabilities, who face increased risks of social isolation and poor mental health. This study aimed to examine the effects of interpersonal relationships on subjective health and mental health among individuals with disabilities.

**Methods:**

We analysed nationally representative panel data of economically active individuals with disabilities in South Korea. Generalized estimating equation models were applied to assess the associations between interpersonal relationship scores and health outcomes, adjusting for sociodemographic and behavioural factors.

**Results:**

Compared with participants with the highest interpersonal relationship scores (≥25 points), those with the lowest scores (≤14 points) had significantly higher odds of reporting poor subjective health (odds ratio [OR] 1.986 [95% confidence interval {CI} 1.392 to 2.832], p<0.0001), lower happiness (–0.913 points [95% CI 0.840 to 0.993], p=0.034) and greater odds of experiencing depression (OR 1.959 [95% CI 1.179 to 3.255], p=0.009).

**Conclusions:**

These findings highlight the importance of social connectedness as a key determinant of mental and subjective health among people with disabilities in Korea.

## Introduction

Improving the quality of life for people with disabilities has emerged as a core objective in international and national policy.^1,2^ Various structural disadvantages, such as physical limitations, social stigma, accessibility barriers and economic constraints, pose major threats to the health and well-being of individuals with disabilities.^3^ While previous health research has predominantly focused on objective indicators, including physical functioning and access to healthcare,^4,5^ recent studies have increasingly highlighted the importance of subjective measures such as self-rated health, mental well-being and experiences of depression.^6,7^ These subjective indicators provide a more comprehensive and realistic reflection of individuals’ lives, often referred to as the ‘lived experience’ of disability,^8^ and can serve as practical benchmarks for policy interventions.

Many earlier studies have focused on how participation in economic activities and job satisfaction impact the health and quality of life of people with disabilities.^9,10^ Employment serves not only as a means of income generation but also deeply influences mental health by fulfilling self-actualization needs and social roles,^11^ especially considering that work occupies a significant portion of an individual's waking hours across many phases of life.

Factors such as employment stability, work environment and job satisfaction have consistently been reported to significantly affect self-rated health and mental well-being.^12^ These findings suggest that improving job environments and expanding employment opportunities could be crucial in enhancing health equity among people with disabilities.^13^

Given the structural challenges that prevent many people with disabilities from engaging in stable economic activities, focusing solely on employment-centred strategies may be insufficient to fully explain or improve their health and well-being. Particularly for individuals facing difficulties entering or remaining in the labour market, broader and more everyday life factors—such as interpersonal relationships and informal social capital—may exert a greater influence on their well-being. Reflecting this, recent research and policy trends increasingly emphasize social integration and psychological well-being in addition to physical independence.^14^ Numerous previous studies, both in South Korea and internationally, have reported that higher levels of interpersonal relationships are associated with improved mental health and subjective well-being.^15–17^ Social relationships serve as a foundation for emotional support, a sense of belonging and the development of self-esteem,^16^ and they also function as buffering factors in stressful situations.^17^ However, empirical research focusing on social relationship variables among people with disabilities remains relatively limited. Most existing studies have concentrated on variables such as employment, income and acceptance of disability, while informal factors like interpersonal relationships have often been excluded from analysis or treated only superficially.^18,19^ In particular, few studies have quantitatively assessed the level of interpersonal skills or perceptions and analysed their associations with health and happiness.^20^

People with disabilities are more likely to experience social exclusion due not only to physical limitations but also to relationship breakdowns, isolation and stigma.^21^ In such circumstances, interpersonal relationships serve as critical resources for emotional recovery and daily adaptation, extending beyond mere social interactions.^22^ Networks comprising family, friends and local communities may sometimes function as stronger psychological support systems than employment itself,^23^ and they are particularly vital for individuals experiencing stress and mental health challenges. Nevertheless, interpersonal relationships have often been regarded as personal attributes and thus excluded from policy considerations.^24^ This oversight can be observed in both international and national disability policy frameworks, which tend to emphasize economic or medical needs over informal social dynamics.^25^ Given this gap, it is now essential to empirically demonstrate the importance of these informal networks and to incorporate them into policy design.

Accordingly, this study utilizes data from the second wave (2016–2018) of the Panel Survey of Employment for the Disabled (PSED) to examine how levels of interpersonal relationships affect self-rated health, mental happiness and experiences of depression among economically active individuals with disabilities. The 2016–2018 second wave data of the PSED were selected because they contain comprehensive items on social relationships and mental health, enabling a more integrated analysis of interpersonal factors and well-being. By supplementing the traditional employment-centred health determinant framework, this study aims to shed light on how everyday, relationship-based psychological and social resources influence the lives of people with disabilities. While prior studies have primarily focused on employment status or income as health determinants among people with disabilities, this study aims to extend the scope of analysis by focusing on interpersonal relationships as a core component of informal social capital. Based on prior evidence, we hypothesize that lower levels of interpersonal relationships will be associated with poorer subjective health, lower happiness in mental health and a greater likelihood of experiencing depression. Therefore, this study aims to examine the associations between interpersonal relationship levels and three specific health outcomes: subjective health status, happiness in mental health and experiences of depression among economically active individuals with disabilities.

## Methods

### Study sample and design

This study utilized data from the first (2016), second (2017) and third (2018) surveys of the second wave of the PSED. The PSED is the first nationally representative longitudinal survey of people with disabilities conducted in South Korea, with data collected through computer-assisted personal interviews (CAPIs) targeting registered persons with disabilities nationwide. As of 15 May 2016, a sample of 4577 individuals ages 15–64 y, registered under Article 2 of the Welfare of Persons with Disabilities Act, was selected. The sample was stratified to account for diversity in age and type of disability and individuals engaged in or seeking to engage in economic activities were oversampled. The survey collected information on demographics (such as gender, age, education level, type and grade of disability), economic activity and personal and environmental factors.

**Figure 1.**
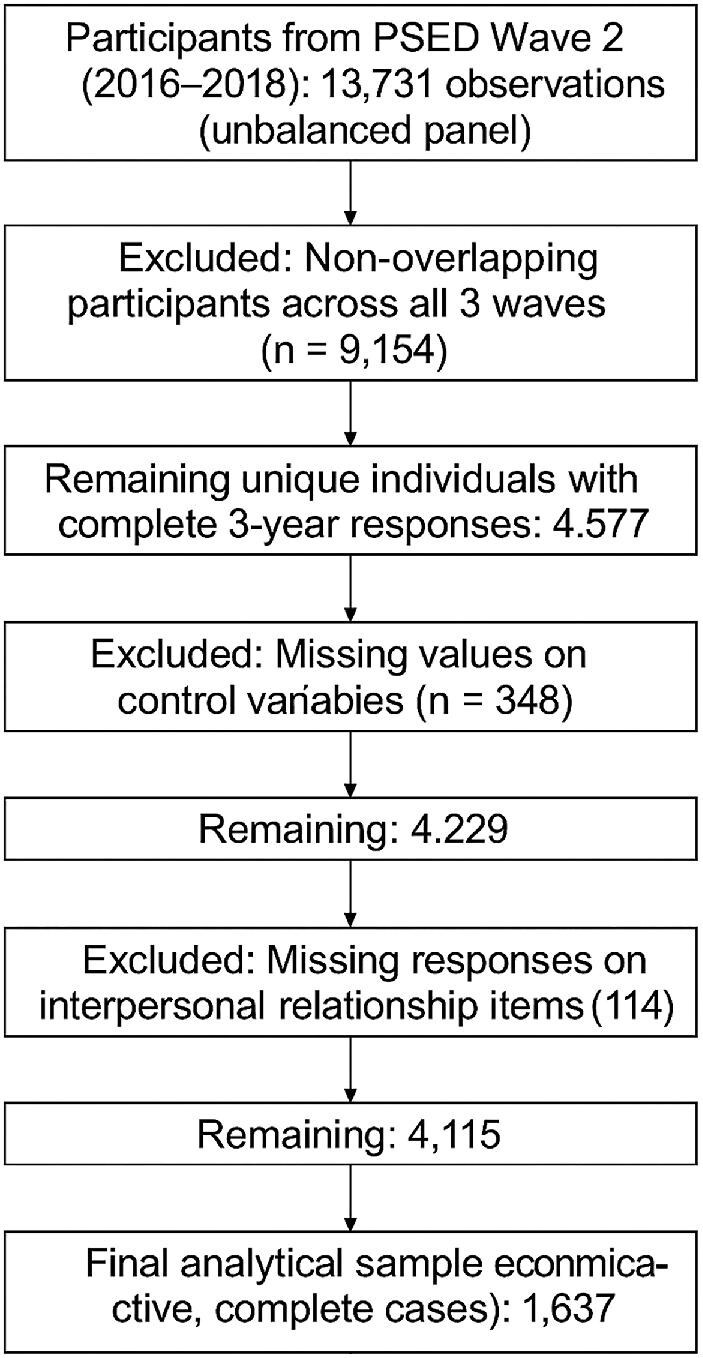
Sample selection flow diagram.

The 2016–2018 second wave data were selected because they include consistently measured variables on social relationships, subjective health and mental health outcomes across all three years. For this study, we extracted economically active individuals who responded to all key variables without missing data across the three waves, resulting in a final sample of 1637 participants. The stepwise exclusion process used to identify the final analytical sample is illustrated in Figure 1, which shows the number of participants retained at each stage of filtering. The PSED was conducted by the Korea Employment Agency for Persons with Disabilities and the original survey received ethical approval from the Institutional Review Board of the Korea Research Institute for Vocational Education and Training (KIVET). Detailed information about the sampling design, questionnaires and data collection methods of the PSED can be found in the official documentation provided by the Korea Employment Agency for Persons with Disabilities (https://www.kead.or.kr).

### Independent variables

#### Overall perception of interpersonal relationships

In this study, the main independent variable was the overall perception of interpersonal relationship ability. This was assessed using seven specific items: (1) ‘I easily make friends’, (2) ‘I initiate conversations before others talk to me’, (3) ‘I am a good listener when my friends share their concerns’, (4) ‘I often compliment others’, (5) ‘I can express my opinion without fighting when I have a different opinion’, (6) ‘I can stay calm without losing my temper when someone gets angry at me’ and (7) ‘I try to resolve issues through dialogue when conflicts arise with my friends’.

Each item was rated on a 4-point Likert scale: 1 (strongly disagree), 2 (disagree), 3 (agree) and 4 (strongly agree). Additionally, participants were asked to rate their overall interpersonal relationship ability through the question, ‘How would you evaluate your overall interpersonal relationship ability?’ Responses were categorized as 1 (very poor), 2 (poor), 3 (good) and 4 (very good).

### Dependent variables

The dependent variables in this study were subjective health status and mental health status. Mental health status was assessed using two items: happiness and depression. Subjective health status was assessed using self-rated health.

#### Self-rated health

Subjective health status was measured using the question, ‘How do you usually perceive your health?’ Responses of ‘very poor’ and ‘poor’ were classified as ‘poor’, whereas responses of ‘good’ and ‘very good’ were classified as ‘good’. This variable was treated as a binary outcome and analysed using logistic regression.

#### Level of happiness in mental health

Happiness was measured using a single question: ‘How happy do you currently feel in terms of your mental health?’ Responses were recorded on a continuous scale ranging from 0 (not happy at all) to 10 (very happy) and the variable was treated as a continuous outcome in the analysis. The use of a 0–10 scale is a standard method for assessing subjective well-being, allowing for meaningful variation in self-perceived happiness levels.

#### Depression experience in the past year

Depression was assessed by the question: ‘Have you experienced depression during the past year?’, which was measured using a binary response format (yes/no). This item was treated as a binary outcome variable, as its structure reflects a diagnostic screening approach commonly used in population-based health surveys.

#### Control variables

The following variables were included as control variables in this study.

Demographic variables included gender (male, female), age group (15–29, 30–39, 40–49, 50–59, 60–66), residential area (metropolitan, urban, rural) and marital status (single, married, divorced, separated, widowed). Health behaviour variables included smoking status (non-smoker, former smoker, current smoker), drinking status (non-drinker, former drinker, current drinker) and stress level (low, moderate, high). Disability-related variables included severity of disability (severe: grades 1–3; mild: grades 4–6) and type of disability (physical disability, other disabilities).

#### Analytical approach and statistics

To examine the associations between interpersonal relationships and self-rated health, happiness in mental health and experiences of depression, χ^2^ tests and generalized estimating equation models were employed. All analyses were conducted using a two-tailed test with a significance level set at p<0.0001. Statistical analyses were performed using SAS version 9.4 (SAS Institute, Cary, NC, USA).

## Results

### Prevalence of self-rated health and happiness

The distribution of subjective health status and happiness among the study participants is presented in Table 1. Among the total participants, 28.8% (471 individuals) reported poor subjective health status.

**Table 1.** General characteristics of subjects included for analysis at baseline.

	Total		Self-rated health					Depression					Happy		
			Poor		Good			Yes		No					
Characteristics	*n*	%	*n*	%	*n*	%	p-Value	*n*	%	*n*	%	p-Value	Mean	SD	p-Value
Interpersonal relationship							<0.0001					0.061			0.0008
≤14	110	6.74	43	39.1	67	60.9		18	16.4	92	83.6		6.709	9.009	
15–19	464	28.41	161	34.7	303	65.3		58	12.5	406	87.5		6.286	4.536	
20–24	853	52.24	222	26.0	631	74.0		78	9.1	775	90.9		6.548	1.448	
≥25	206	12.61	45	21.8	161	78.2		22	10.7	184	89.3		7.694	6.536	
Gender							0.0002					0.3866			0.4779
Male	1231	75.38	326	26.5	905	73.5		128	10.4	1103	89.6		6.670	4.799	
Female	402	24.62	145	36.1	257	63.9		48	11.9	354	88.1		6.502	1.473	
Age (years)							<0.0001					0.7114			0.0377
15–29	241	14.76	50	20.8	191	79.3		26	10.8	215	89.2		7.327	8.561	
30–39	508	31.11	98	19.3	410	80.7		47	9.3	461	90.8		6.606	1.385	
40–49	527	32.27	162	30.7	365	69.3		60	11.4	467	88.6		6.628	4.286	
50–59	248	15.19	105	42.3	143	57.7		29	11.7	219	88.3		6.181	1.517	
60–66	109	6.67	56	51.4	53	48.6		14	12.8	95	87.2		6.220	1.455	
Residential region							0.335					0.0001			0.8702
Metropolitan area	345	21.13	105	30.4	240	69.6		49	14.2	296	85.8		6.669	5.241	
Metropolitan cities	423	25.90	130	30.7	293	69.3		23	5.4	400	94.6		6.491	1.392	
Other provinces	865	52.97	236	27.3	629	72.7		104	12.0	761	88.0		6.680	4.681	
Marital status							<0.0001					0.0001			0.0236
Married/living together	898	54.99	242	27.0	656	73.1		78	8.7	820	91.3		6.767	3.389	
Single	547	33.50	142	26.0	405	74.0		62	11.3	485	88.7		6.674	5.801	
Divorced/widowed/separated	188	11.51	87	46.3	101	53.7		36	19.2	152	80.9		5.840	1.442	
Smoking status							0.2995					0.038			0.0357
Current smoker	469	28.72	127	27.1	342	72.9		64	13.7	405	86.4		6.234	1.503	
Former smoker	367	22.47	117	31.9	250	68.1		40	10.9	327	89.1		6.833	5.048	
Never smoked	797	48.81	227	28.5	570	71.5		72	9.0	725	91.0		6.767	4.849	
Alcohol consumption							<0.0001					0.372			0.7009
Current drinker	948	58.05	228	24.1	720	76.0		103	10.9	845	89.1		6.533	3.351	
Former drinker	258	15.80	105	40.7	153	59.3		33	12.8	225	87.2		6.864	5.944	
Never drank	427	26.15	138	32.3	289	67.7		40	9.4	387	90.6		6.700	4.715	
Stress							<0.0001					<.0001			0.0135
Yes	722	44.21	145	20.1	577	79.9		29	4.0	693	96.0		6.965	3.689	
No	911	55.79	326	35.8	585	64.2		147	16.1	764	83.9		6.363	4.599	
Disability grade							0.1164					0.1642			0.4548
1–3	421	25.78	134	31.8	287	68.2		53	12.6	368	87.4		6.627	4.758	
4–6	1212	74.22	337	27.8	875	72.2		123	10.2	1089	89.9		6.630	4.033	
Type of disability							0.8209					0.0507			0.8853
Physical	929	56.89	270	29.1	659	70.9		88	9.5	841	90.5		6.636	3.360	
Other	704	43.11	201	28.6	503	71.5		88	12.5	616	87.5		6.620	5.161	
Total	1633	100	471	28.8	1162	71.2		176	10.8	1457	89.2		6.629	4.230	

To facilitate interpretation, interpersonal relationship scores were categorized into quartile-based groups. In the group with the lowest scores (≤14 points, bottom quartile), 39.1% (43 individuals) reported poor health, whereas in the group with the highest scores (≥25 points, top quartile), the proportion was 21.8% (45 individuals). These labels were applied to improve the clarity of comparison across score groups.

Regarding happiness, the mean score among all participants was 6.63 (standard deviation [SD] 4.23). Participants with the lowest interpersonal relationship scores (≤14 points) had a mean happiness score of 6.71 (SD 9.01), while those with the highest scores (≥25 points) had a mean score of 7.69 (SD 6.54).

### Association between interpersonal relationships and self-rated health and depression

Table 2 presents the results examining the associations between interpersonal relationship scores, self-rated health and experiences of depression. Participants with lower interpersonal relationship scores had significantly higher odds of reporting poor self-rated health (p<0.0001 for ≤14 points, p=0.001 for 15–19 points) and experiencing depression (p=0.009 for ≤14 points, p=0.046 for 15–19 points) compared with those in the highest score group (≥25 points).

**Table 2.** Association between interpersonal relationship scores and self-rated health.

	Self-rated health			Depression		
Characteristics	OR	95% CI	p-Value	OR	95% CI	p-Value
Interpersonal relationship						
≤14	1.986	1.392 to 2.832	0.000	1.959	1.179 to 3.255	0.009
15–19	1.516	1.187 to 1.935	0.001	1.472	1.006 to 2.152	0.046
20–24	1.144	0.910 to 1.439	0.248	0.984	0.683 to 1.417	0.930
≥25	1.000			1.000		
Gender						
Male	0.739	0.609 to 0.897	0.002	0.546	0.403 to 0.739	<0.0001
Female	1.000			1.000		
Age (years)						
15–29	0.156	0.110 to 0.223	<0.0001	0.833	0.464 to 1.494	0.539
30–39	0.218	0.165 to 0.287	<0.0001	1.021	0.625 to 1.669	0.933
40–49	0.357	0.277 to 0.461	<0.0001	1.148	0.719 to 1.834	0.563
50–59	0.560	0.429 to 0.730	<0.0001	1.386	0.856 to 2.242	0.184
60–66	1.000			1.000		
Residential region						
Metropolitan area	0.926	0.772 to 1.110	0.406	0.847	0.646 to 1.111	0.232
Metropolitan city	1.075	0.913 to 1.265	0.384	0.464	0.345 to 0.623	<0.0001
Other provinces	1.000			1.000		
Marital status						
Married/living together	0.517	0.421 to 0.635	<0.0001	0.395	0.294 to 0.531	<0.0001
Single	0.785	0.608 to 1.013	0.063	0.742	0.517 to 1.064	0.104
Divorced/widowed/separated	1.000			1.000		
Smoking status						
Current smoker	1.274	1.038 to 1.563	0.021	1.774	1.289 to 2.441	0.000
Former smoker	1.115	0.904 to 1.375	0.309	1.237	0.876 to 1.747	0.228
Never smoked	1.000			1.000		
Alcohol consumption						
Current drinker	0.679	0.560 to 0.823	<0.0001	1.218	0.895 to 1.658	0.210
Former drinker	1.110	0.890 to 1.385	0.354	1.569	1.102 to 2.235	0.013
Never drank	1.000			1.000		
Stress						
Yes	0.457	0.397 to 0.527	<0.0001	0.282	0.219 to 0.364	<0.0001
No	1.000			1.000		
Disability grade						
1–3	1.349	1.139 to 1.598	0.001	1.152	0.889 to 1.493	0.283
4–6	1.000			1.000		
Type of disability						
Physical	1.322	1.135 to 1.539	0.000	0.852	0.673 to 1.077	0.180
Other	1.000			1.000		
Year						
2016	1.220	1.035 to 1.439	0.018	1.609	1.243 to 2.083	0.000
2017	0.913	0.770 to 1.081	0.291	1.087	0.824 to 1.434	0.554
2018	1.000			1.000		

In addition, several covariates were significantly associated with the outcomes. These included gender (p=0.002 for self-rated health, p<0.0001 for depression), age group (p<0.0001 across younger age categories for self-rated health), marital status (p<0.0001), smoking and drinking status (p=0.021 and p<0.0001, respectively), perceived stress levels (p<0.0001) and survey year (p=0.018 and p=0.000 in 2016).

### Association between interpersonal relationships and self-rated health and happiness

Table 3 presents the results of the analysis examining the associations between interpersonal relationship scores, subjective health status and happiness in mental health. The analysis adjusted for all confounding factors.

**Table 3.** Association between interpersonal relationship scores and experiences of depression.

	Self-rated health			Happy		
Characteristics	OR	95% CI	p-Value	B	95% CI	p-Value
Interpersonal relationship						
≤14	1.986	1.392 to 2.832	0.000	0.913	0.840 to 0.993	0.034
15–19	1.516	1.187 to 1.935	0.001	0.865	0.820 to 0.912	<0.0001
20–24	1.144	0.910 to 1.439	0.248	0.912	0.870 to 0.956	0.000
≥25	1.000			REF		
Gender						
Male	0.739	0.609 to 0.897	0.002	1.024	0.979 to 1.071	0.307
Female	1.000			REF		
Age (years)						
15–29	0.156	0.110 to 0.223	<0.0001	1.138	1.045 to 1.239	0.003
30–39	0.218	0.165 to 0.287	<0.0001	1.078	1.005 to 1.157	0.037
40–49	0.357	0.277 to 0.461	<0.0001	1.070	0.999 to 1.146	0.055
50–59	0.560	0.429 to 0.730	<0.0001	1.020	0.948 to 1.098	0.593
60–66	1.000			REF		
Residential region						
Metropolitan area	0.926	0.772 to 1.110	0.406	0.964	0.924 to 1.005	0.087
Metropolitan city	1.075	0.913 to 1.265	0.384	0.973	0.936 to 1.012	0.172
Other provinces	1.000			REF		
Marital status						
Married/living together	0.517	0.421 to 0.635	<0.0001	1.110	1.048 to 1.177	0.000
Single	0.785	0.608 to 1.013	0.063	1.070	0.999 to 1.145	0.053
Divorced/widowed/separated	1.000			REF		
Smoking status						
Current smoker	1.274	1.038 to 1.563	0.021	0.950	0.906 to 0.996	0.035
Former smoker	1.115	0.904 to 1.375	0.309	1.017	0.970 to 1.066	0.481
Never smoked	1.000			REF		
Alcohol consumption						
Current drinker	0.679	0.560 to 0.823	<0.0001	0.977	0.934 to 1.022	0.316
Former drinker	1.110	0.890 to 1.385	0.354	1.005	0.953 to 1.060	0.847
Never drank	1.000			REF		
Stress						
Yes	0.457	0.397 to 0.527	<0.0001	1.072	1.038 to 1.107	<0.0001
No	1.000			REF		
Disability grade						
1–3	1.349	1.139 to 1.598	0.001	0.970	0.931 to 1.010	0.139
4–6	1.000			REF		
Type of disability						
Physical	1.322	1.135 to 1.539	0.000	0.976	0.942 to 1.011	0.174
Other	1.000			REF		
Year						
2016	1.220	1.035 to 1.439	0.018	0.998	0.960 to 1.038	0.931
2017	0.913	0.770 to 1.081	0.291	1.021	0.982 to 1.062	0.287
2018	1.000			REF		

REF: reference.

Lower interpersonal relationship scores were significantly associated with higher odds of reporting poor health. Participants scoring ≤14 points had an OR of 1.986 (95% CI 1.392 to 2.832, p<0.0001), scores between 15–19 points had an OR of 1.516 (95% CI 1.187 to 1.935, p=0.001) and scores between 20–24 points had an OR of 1.144 (95% CI 0.910 to 1.439, p=0.248).

In terms of happiness, participants with the lowest interpersonal relationship scores had an average of 0.913 points lower happiness scores than those with the highest scores (95% CI 0.840 to 0.993, p=0.034). Scores of 15–19 points were associated with 0.865 points lower happiness scores (95% CI 0.820 to 0.912, p<.0001) and scores of 20–24 points were associated with 0.912 points lower happiness scores (95% CI 0.870 to 0.956, p=0.000).

## Discussion

This study analysed the effects of interpersonal relationship levels on subjective health status, mental health–related happiness and experiences of depression among economically active individuals with disabilities. Utilizing nationally representative panel data, the findings confirmed that social connectedness is a crucial determinant of both physical and mental health for people with disabilities.

The main findings indicated that lower levels of interpersonal relationships were significantly associated with a higher likelihood of poor subjective health status, lower happiness in mental health and a greater probability of experiencing depression. These associations remained statistically significant even after adjusting for demographic, health status and health behaviour variables, supporting existing theoretical discussions about the role of social relationships in mental health and well-being.^26,27^ These results are consistent with previous findings that emphasize the role of social capital in buffering psychological distress and improving perceived health. However, the strong effect size observed in this study suggests that interpersonal relationships may be an even more critical factor for people with disabilities, particularly due to their increased risk of social exclusion and stigma.

This study highlights the critical influence of informal social capital, particularly interpersonal relationships, on the health and mental well-being of people with disabilities. Specifically, individuals with the lowest interpersonal relationship scores (≤14 points) were nearly twice as likely to report poor subjective health status compared with those with the highest scores (≥25 points). This finding supplements previous perspectives that focused primarily on employment status or job satisfaction as health determinants,^28,29^ emphasizing the importance of relational and emotional resources. To fully understand the complex structure of health among people with disabilities, it is necessary to consider interpersonal and emotional resources alongside employment conditions.

Lower interpersonal relationship levels were also closely associated with a higher likelihood of experiencing depression. Social isolation and disconnection are recognized as major risk factors for mental health problems such as depression,^30^ and these risks may be even more pronounced among people with disabilities, who are vulnerable to social stigma and discrimination. These findings suggest that the construction of social support systems is essential, moving beyond an individual-focused interpretation of psychological vulnerability to address the broader social context. Individuals with weak social networks may exhibit lower resilience to stress, which may consequently deteriorate mental health over time.^31,32^

Happiness in mental health was also found to be significantly influenced by interpersonal relationships. In this study, happiness in mental health was measured using a single-item question from the PSED, asking participants, ‘How happy do you currently feel in terms of your mental health?’ on a scale from 0 (not happy at all) to 10 (very happy). Participants with lower interpersonal relationship scores consistently reported lower levels of happiness, consistent with previous studies emphasizing the importance of social relationships for emotional stability and subjective life satisfaction.^33,34^ This study demonstrated that such trends persist among people with disabilities. Moreover, happiness should not be viewed merely as a measure of temporary mood but as a comprehensive indicator reflecting the overall quality of life.^35^ Therefore, policies and programs aimed at improving mental well-being among people with disabilities should prioritize the restoration of relational stability and the sense of belonging, beyond psychotherapy or counselling alone.^36^

This study provides several implications for health policy and practice related to people with disabilities. In addition to strengthening job capabilities and expanding employment opportunities, strategies to promote the health of people with disabilities should prioritize the formation and maintenance of social networks. Early identification of individuals with vulnerable interpersonal relationships and the provision of emotional support through community-based interaction programs could serve as effective interventions. Programs such as peer mentoring, community groups and local self-help initiatives may facilitate natural relationship building and create environments that foster psychological support. Furthermore, relational aspects should be incorporated into service delivery systems aimed at enhancing the health of people with disabilities. For instance, emotional relationship building could be integrated as a goal within case management services at public health centres and welfare institutions, and social support interventions could be embedded into existing home healthcare programs. Mental health promotion and social network expansion should be understood as mutually reinforcing efforts, not separate domains.

Despite these contributions, this study has several limitations. First, although panel data were used, the analysis was cross-sectional in nature, limiting the ability to infer causality. Second, interpersonal relationships were measured using self-reported items, which may not fully reflect the actual quality or depth of those relationships. Third, the study focused exclusively on economically active individuals with disabilities, which may limit the generalizability of the findings to those not participating in economic activities. Finally, happiness was assessed using a single-item measure, which may not capture the multidimensional nature of psychological well-being. Future studies should consider longitudinal designs and more comprehensive measures of mental well-being to build upon these findings. Nevertheless, this study makes important contributions by empirically confirming the association between interpersonal relationships and the health and well-being of people with disabilities. It expands the perspective of previous approaches that primarily focused on structural or economic factors and provides new insights into the psychosocial dimensions of health.

### Policy implications and recommendations

Based on the findings of this study, we propose several policy implications centred on the importance of interpersonal relationships for people with disabilities. First, future research and interventions should consider relationship-based strategies tailored to the type of disability, gender and age group. Our findings suggest that the quality of interpersonal relationships is significantly associated with both subjective health and mental health outcomes. Targeted programs that foster social inclusion and positive relational experiences could serve as protective factors, particularly for individuals who may be structurally excluded from formal economic participation. Second, it is essential to recognize the ‘right to form and maintain relationships’ as a basic component of health equity for people with disabilities. Health and welfare policies should support environments where interpersonal connection is encouraged, such as through inclusive community spaces, peer-support programs and accessible digital communication platforms.

These recommendations are supported by recent international studies. Al-Shaer et al.^37^ found that emotional support significantly buffers the negative effects of stress and anxiety on quality of life among students with disabilities. Similarly, a study conducted in Saudi Arabia among sports club participants with disabilities demonstrated that higher levels of social inclusion were associated with better mental health outcomes, including lower depression and anxiety levels.^38^ Taken together, these findings reinforce the urgent need for disability policies that explicitly incorporate social connectedness as a core determinant of both physical and mental health.

## Data Availability

The data underlying this article are available from the Korea Employment Agency for Persons with Disabilities (https://www.kead.or.kr). The PSED dataset is accessible for research purposes upon request and approval from the agency.
